# Effects of lifestyle intervention in *BRCA1/2* mutation carriers on nutrition, BMI, and physical fitness (LIBRE study): study protocol for a randomized controlled trial

**DOI:** 10.1186/s13063-016-1504-0

**Published:** 2016-07-29

**Authors:** Marion Kiechle, Christoph Engel, Anika Berling, Katrin Hebestreit, Stephan C. Bischoff, Ricarda Dukatz, Michael Siniatchkin, Katharina Pfeifer, Sabine Grill, Maryam Yahiaoui-Doktor, Ellen Kirsch, Uwe Niederberger, Ute Enders, Markus Löffler, Alfons Meindl, Kerstin Rhiem, Rita Schmutzler, Nicole Erickson, Martin Halle

**Affiliations:** 1 Department of Gynecology and Center for Hereditary Breast and Ovarian Cancer, Klinikum Rechts der Isar, Technical University Munich (TUM) and Comprehensive Cancer Center Munich (CCCM), Ismaninger Str. 22, 81675 Munich, Germany; 2 Institute for Medical Informatics, Statistics and Epidemiology, University of Leipzig, Haertelstrasse 16-18, 04107 Leipzig, Germany; 3 Department of Prevention and Sports Medicine, Klinikum rechts der Isar, Technical University Munich (TUM), Ismaninger Str. 22, 81675 Munich, Germany; 4 Institute for Nutritional Medicine, University Hohenheim, Fruwirthstr. 12, 70593 Stuttgart, Germany; 5 Institute for Medical Psychology and Sociology, University Hospital Schleswig-Holstein, Campus Kiel, Preusserstr. 1-9, 24105 Kiel, Germany; 6 Center for Hereditary Breast and Ovarian Cancer, University Hospital Cologne, Kerpener Str. 34, 50931 Cologne, Germany; 7 Else Kröner-Fresenius Prevention Center, Klinikum rechts der Isar, Technical University Munich (TUM), Ismaninger Str. 22, 81675 Munich, Germany

**Keywords:** *BRCA1*, *BRCA2*, Hereditary breast cancer, Hereditary ovarian cancer, Lifestyle intervention

## Abstract

**Background:**

Women with highly penetrant *BRCA* mutations have a 55–60 % lifetime risk for breast cancer and a 16–59 % lifetime risk of developing ovarian cancer. However, penetrance differs interindividually, indicating that environmental and behavioral factors may modify this risk. It is well documented that the risk for sporadic breast cancer disease can be modified by changing lifestyle factors that primarily include physical activity, dietary habits, and body weight. It can thus be hypothesized that the modification of these lifestyle factors may also influence the incidence and progression of cancer in *BRCA* mutation carriers.

**Methods/design:**

This multicenter, interdisciplinary, prospective, two-armed, randomized (1:1) controlled trial aims to enroll a minimum of 600 *BRCA1* and *BRCA2* mutation carriers to partake in either a lifestyle intervention or usual care. The study primarily aims to demonstrate an improvement of nutritional behavior (adherence to the Mediterranean diet), body mass index, and physical fitness. Furthermore, the effects on quality of life, stress coping capacity, breast cancer incidence, and mortality will be investigated. The intervention group (IG) will receive a structured lifestyle intervention over 12 months, whereas the control group (CG) will only receive information regarding a healthy lifestyle. During the first 3 months, women in the IG will receive structured, individualized, and mainly supervised endurance training with a minimum of 18 MET-h physical activity per week and nutrition education based on the Mediterranean diet. Over the following 9 months, IG monthly group training sessions and regular telephone contacts will motivate study participants. The CG will receive one general training session about healthy nutrition in accordance with the recommendations of the German Society of Nutrition (standard of care in Germany) and the benefits of regular physical activity on health status. At randomization and subsequent time points (3 and 12 months), cardiopulmonary fitness will be assessed by spiroergometry, and nutritional and psychological status will be assessed by validated questionnaires, interviews, and clinical examinations.

**Discussion:**

As data on the role of lifestyle intervention in women with a hereditary risk for breast and ovarian cancer are currently lacking, this study will be of major importance from a scientific, as well as a practical advice viewpoint. It will investigate the optimal strategy to improve physical fitness, nutritional status, and psychological factors such as quality of life, perceived stress, optimism, as well as incidence and outcome of cancer in this selected group of women at high risk. If the study indicates a positive long-term outcome, a structured lifestyle intervention program could be added to health care prevention strategies for *BRCA1* and *BRCA2* mutation carriers.

**Trial registration:**

ClinicalTrials.gov: NCT02516540. Registered on 22 July 2015.

**Electronic supplementary material:**

The online version of this article (doi:10.1186/s13063-016-1504-0) contains supplementary material, which is available to authorized users.

## Background

Women with highly penetrant *BRCA* mutations have a 55–60 % risk for breast cancer and a 16–59 % risk of developing ovarian cancer [1, 2]. However, since penetrance rates are not 100 %, it can be postulated that risk-modulating factors do exist. It can be shown that the risk of development of cancer in gene carriers may be influenced through genetic factors (polymorphisms) as well as exogenous factors such as number of pregnancies, year of birth, and physical activity during youth [3, 4]. The risk for breast cancer is lower if gene carriers were born before 1940, have given birth, or were physically active during their youth [5, 6].

The risk for developing sporadic breast cancer is considerably influenced by physical activity, nutrition, and body weight factors which also affect disease progression. Likewise, it has been demonstrated in several prospective studies that regular physical activity can significantly reduce breast cancer incidence in postmenopausal and premenopausal women, the risk being reduced on average by 25 % [7]. Furthermore, the risks of recurrence and mortality in women with breast cancer are reduced by 50 % if they engage in regular physical activity [8]. Further advantages of physical activity include a gain in the quality of life, increased fitness, and better tolerance of chemotherapy [9]. Nutrition also influences the risk for breast cancer. Obesity and weight gain increase the risk of breast cancer in both pre- and postmenopausal subjects [10]. A weight gain of more than 20 kg after the age of 18 doubles the risk for breast cancer. Furthermore, women with a body mass index (BMI) of >30 kg/m^2^ have a greater risk of developing distant metastases and of dying of breast cancer [11]. In a prospective study of patients with sporadic breast cancer who were given adjuvant standard therapy, a calorie- and fat-reduced nutrition program led to a significant improvement in recurrence rate [12]. Additionally, in the prospective PREDIMED prevention trial, women in the Mediterranean diet arm supplemented with extra-virgin olive oil had a 68 % lower risk for breast cancer compared with the controls [13].

Further risk factors for breast cancer include depression, a pessimistic outlook on life, and problems of coping with stress [14, 15]. It was shown that physical activity has a favorable influence on stress management and depression. Many studies have convincingly documented the great significance of an optimistic life perspective for different psychological and somatic disorders [16]. A positive correlation was shown between an optimistic outlook on life and psychological well-being, health, stress reduction, and mortality, as well as a quicker recovery rate [17–19].

So far, no studies exist in this context on women with hereditary breast cancer or women with a deleterious *BRCA* mutation. Even retrospective data are rare. There is only one publication on this subject by Manders in 2011 [20], which reports on an association between increased body weight and an increased risk for breast cancer in *BRCA1/2* mutation carriers.

We therefore aim to examine whether a lifestyle intervention in the form of structured physical endurance training and nutrition education, emphasizing the Mediterranean dietary pattern, will lead to an improvement of nutritional behavior (adherence to the Mediterranean diet), BMI, physical fitness, quality of life, and optimistic outlook on life, and cause a significant reduction of the perceived stress. Secondary aims of the study are to investigate whether the intervention will lead to a reduction of breast cancer incidence and breast cancer mortality in *BRCA1* and *BRCA2* mutation carriers.

## Design/methods

### Trial design

**Fig. 1:**
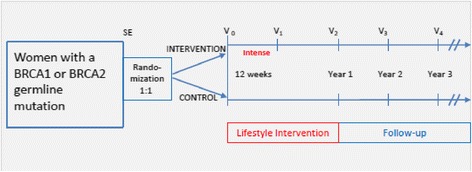
Design of the trial

The study is a multicenter, prospective, two-armed randomized (1:1) controlled clinical trial. The primary aim of the study is to evaluate whether a structured, one-year exercise program combined with a Mediterranean dietary pattern in *BRCA* mutation carriers will improve BMI, physical fitness (ventilatory threshold VT1 in spiroergometry), and adherence to a Mediterranean diet in the intervention group. Additionally, we will analyze whether the lifestyle intervention has a positive effect on quality of life, perceived stress, optimistic outlook on life, breast cancer incidence, and mortality. The study design is outlined in Fig. 1 and includes six visits (SE: study entry, V0: start of intervention, V1: 3 months after start of intervention, V2: 12 months after start of intervention, V3: 24 months after start of intervention, and V4: 36 months after start of intervention). The Standard Protocol Items: Recommendations for Interventional Trials (SPIRIT) checklist and flow diagram are available as Additional files 1 and 2.

### Participants

The goal is to recruit a minimum of 600 patients. Recruitment and study conduct take place at 18 centers of the German Consortium for Hereditary Breast and Ovarian Cancer (GC-HBOC). Table 1 details the study’s inclusion and exclusion criteria.

**Table 1:** Study population: inclusion and exclusion criteria

Inclusion criteria (all criteria must apply): • Women with a *BRCA1* or *BRCA2* germline mutation • Age over 18 years • Written informed consent
Exclusion criteria: • Ongoing chemo- or radiation therapy (recruitment is possible 6 weeks after completing therapy) • Metastatic tumor disease • Expectancy of life <3 years • Limited cardiovascular and lung diseases (unstable CHC, heart failure stage IV, chronic obstructive pulmonary disease GOLD IV, maximal blood pressure at rest >160/100 mmHg) • Significant orthopedic problems not allowing exercise • Serious diseases not allowing a participation in group interventions (e.g., psychiatric or internal ailment) • Karnofsky index <60 % • Women with an exercise capacity <50 watts • Food allergies not allowing consumption of a Mediterranean dietary pattern • Vegans • BMI <15 kg/m^2^ • Pregnancy • Insufficient knowledge of the German language • Lack of compliance • Current participation in other lifestyle intervention trials

*CHC* Coronary Heart Condition

### Intervention group

A lifestyle intervention program lasting 12 months is adopted in the intervention group. The program is applied intensively during the first 3 months and is maintained and monitored for the following 9 months through monthly contacts and meetings. The lifestyle intervention program comprises the measures described in the following paragraphs.

#### Physical activity

The LIBRE training program is primarily endurance-oriented training, which is completed during the course of one year. After a mandatory introductory lecture on the theory behind the intervention training, the goal is to increase physical activity to ≥18 MET-h/week (MET = Metabolic Equivalent of Task). This activity level has been correlated consistently with a reduction of morbidity and mortality in patients with breast cancer [8, 19]. This goal should be achieved within the first 12 weeks and maintained throughout the whole study period. Each subject receives an individual training plan, which is continuously adapted according to her fitness status to support this goal. The training is divided into two phases: the intensity of the first phase should be at least 50–60 % of peak oxygen consumption (VO_2_ peak) (initial phase, weeks 1–6), and that of the second phase should be 60–75 % of the VO_2_ peak (optimization phase, weeks 7–12). In the first 12 weeks of the intervention program, training takes place 2 times per week as supervised training and once per week as home-based training (HBT). Afterwards supervised training units are only carried out monthly so that training in phase II (months 4–12) is mainly HBT. Training continues to take place with an intensity of at least 60–75 % of the VO_2_ peak in accordance with the individual training plan.

A record of compliance with the training intervention program as well as with the achieved MET-hours/week is kept in the training diaries (questionnaire on physical activity, V0–V2). Participants are asked to record their daily activities as well as intentional physical activity including intensity and duration of training. The training intensity levels are assessed from spiroergometry and are outlined in the diary, which facilitates training control. Monthly supervised training units (as of V1) offer the opportunity to realign training intensity and discuss any problems with adherence to training. In addition, physical activity is recorded by questionnaires (International Physical Activity Questionnaire, IPAQ) [21, 22] from SE–V4, and physical fitness is assessed by cardiopulmonary exercise testing by spiroergometry (VO_2_ peak) [23] at time points SE, V1, and V2.

#### Nutrition

Within the framework of the LIBRE study, the nutrition intervention is based on the principles of a Mediterranean dietary pattern (MD). Furthermore, obese patients (BMI ≥30 kg/m^2^) are instructed to limit their energy (kilocalorie) intake. Nutrition intervention in the intervention group begins with an intensive 3-month nutrition program during which biweekly nutrition courses led by dietitians take place in groups. The group course includes a cooking class and a guided tour of a supermarket. At the end of the first 3 months, the nutrition courses take place at monthly intervals for the remaining course of the first study year. The main objective of the nutrition intervention is to provide practical nutritional training, which should enable the subjects to achieve a long-term change in their eating habits, replacing former eating habits with the MD. Eating habits are recorded using validated questionnaires (the European Prospective Investigation into Cancer and Nutrition Food Frequency Questionnaire [EPIC-FFQ] and Mediterranean Diet Adherence Screener [MEDAS]) at the time points SE, V1, V2, V3, and V4. Participants of the intervention group additionally received the MEDAS questionnaire at time points V1-6 and V1-9.

#### Psychological support

Psychological support of the intervention group comprises solely an explanation of the psychological data survey questionnaires given to the study participants. Explicit psychological support is not planned. The participants are informed that the objective of the lifestyle study is, among other things, that the lifestyle change including regular physical activity and healthy eating should lead to an improvement in general quality of life, stress reduction, a more optimistic outlook on life, and a better attitude towards endurance training and MD. In order to verify this, the participants receive the questionnaires Trier Inventory for Chronic Stress (TICS), Life Orientation Test-Revised (LOT-R), European Organisation for Research and Treatment of Cancer Quality of Life Questionnaires-Core 30 and BR23 (EORTC QLQ-C30/-BR23), and Evaluation of Physical Activity and Nutrition (“Bewertung koerperlicher Aktivitaet und Ernaehrung” [BKAE]) at different time points (SE, V1, V2, V3, and V4). Advice on psycho-oncological aspects of the LIBRE study is given during the introductory lecture at the beginning of the study. Particular importance is attached to participants giving as detailed information as possible in the survey questionnaires.

Psychological advice given to the study participants should serve to inform the subjects of the significance and improvement of psycho-social lifestyles for the prevention of breast and ovarian cancer, as well as to promote compliance and recognize possible psychological impediments for participation in the study.

### Control group

The control group will receive a mandatory introductory lecture on the positive effects of physical activity on the incidence and prognosis of breast cancer. Afterwards all participants will be given a brochure providing the most important facts on this topic.

Contrary to the intervention group, no training and no physical activity diaries will be provided. Changes in physical activity behavior are measured identically to the interventional group through questionnaires (IPAQ at SE–V4) and examinations of physical capacity (VO_2_ peak) at time points SE, V1, and V2.

Additionally, a dietitian-led nutrition group lesson on healthy eating will be held for the control group. During this lesson the subjects receive general information based on the recommendations of the German Society of Nutrition, which is referred to as “usual care in Germany” in this study. Eating habits will be recorded identically as in the intervention group via validated questionnaires (EPIC-FFQ and MEDAS) at the defined time points SE, V1, V2, V3, and V4.

The psychological guidance of the control group consists of an explanation of the psychological data entry forms which are given to the study participants. An explicit psycho-oncological intervention strategy is also not intended in this group. The participants will be informed that changes in daily routine concerning physical activity and dietary habits among other things should resolve in improvement of quality of life, a reduction of stress, and a more optimistic approach to the future. In order to verify this, the participants will receive questionnaires (TICS, LOT-R, EORTC QLQ-C30/-BR23, and BKAE for registration of changes in attitude towards physical activity and MD) at SE and V1–V4. The psychological information of the control group corresponds to that of the intervention group.

### Measurements

In order to achieve the study goals and to answer the research questions, structured questionnaires and interview sheets, diaries, clinical and instrument-based examinations, as well as blood and stool tests will be used. These are listed and described in the following sections.

#### Medical history

Data will be gathered by means of an interview at study enrollment (SE). In addition to a basic clinical assessment, pre-existing internal and gynecological illnesses, and general risk and protective factors such as body weight, alcohol consumption, nutritional behavior, level of activity, and number of pregnancies will be assessed.

#### Sociodemographics

This will also be collected at SE. The psychosocial and socioeconomic risk and protective factors such as living conditions, job situation, and income level will be assessed.

#### Medical radiation exposure

Data will be gathered by means of an interview at SE. The previous diagnostic and therapeutic radiation exposure will be documented.

#### Study satisfaction questionnaire

This will be handed out at V1, V2, V3, and V4. Questions will capture the subject’s acceptance of the study, their satisfaction with the supervision, an evaluation of the physical activity and nutrition program, and their assessment of the everyday practicality of the intervention program.

#### Cardiovascular assessment

Within this examination at time points SE and V1–V4, the patient’s medical history is taken and a clinical examination with focus on the cardiovascular system will be performed. A resting electrocardiogram (ECG), pulmonary function testing, and blood pressure measurements are obligatory. Resting heart rate and blood pressure are recorded as measuring parameters.

#### Exercise testing/spiroergometry

Spiroergometry is an objective and well-known examination for assessment of the cardiopulmonary system, as it measures the respiratory gases under exercise stress. As the maximal oxygen uptake and the aerobic and anaerobic respiratory capacity are measured, the results enable a precise statement about the maximum cardiopulmonary capacity as well as aerobic and anaerobic capacity. The target parameters VO_2_ peak as well as VO_2_ at ventilatory aerobic and anaerobic thresholds (VT1 and VT2) [23] provide information on the fitness level at visits SE, V1, and V2.

#### International Physical Activity Questionnaire

The International Physical Activity Questionnaire (IPAQ) comprises five areas of activity, which are examined independently of each other [24]. The questionnaire is a simple instrument which can be used to collect internationally comparable data for health-promoting physical activity. The aim is to assess different modes of physical activity performed in daily life. The questionnaire relates to the time which has been spent on physical activity during the last seven days. Activities within the scope of occupational, household, and garden tasks and moving between destinations are included as well. The IPAQ is assessed at all study time points (SE, V1–V4).

#### Examination of physical constitution

The examination of physical constitution comprises the documentation of anthropometric parameters such as height, body weight, waist, hip, and upper arm measurements, skinfold tests, and the bioelectric impedance analysis (BIA) measurement at visits SE, V1, V2, V3, and V4. These measurements serve to record the percentage of body fat, BMI, and level of muscle mass and body water.

#### Blood tests

Within the context of the study, various blood values are determined (Table 2). Blood values are measured in the intervention group as well as in the control group at the time points (SE, V1–V4).

**Table 2:** Blood measurements

Routine blood parameters measured in recruiting centers: • Glutamate pyruvate transaminase (GPT), alanine aminotransferase (ALT) • Total cholesterol • High-density lipoprotein (HDL) cholesterol • Low-density lipoprotein (LDL) cholesterol • Triglycerides • Glucose • Blood cell count • Gamma-glutamyl transferase (GGT)
Blood parameters measured in central or special laboratories: • Insulin • Proencephalin A 119 D159 (Sphingotec GmbH) • Proneurotensin 1–117 (Sphingotec GmbH) • High-sensitivity C-reactive protein (hsCRP) • Omega-3, 6, and 9 fatty acid content in the membrane of erythrocytes

#### Stool tests

The subjects in the intervention group as well as in the control group take stool samples at home at the five time points (SE, V1–V4). A collective microbiome and metabolome analysis is carried out at a later date.

#### EPIC Food Frequency Questionnaire

The EPIC Food Frequency Questionnaire (EPIC-FFQ) [25–27] refers to food consumption during the past year and covers 148 food items. For each item questions are asked concerning the average quantity consumed (predefined servings) and the frequency of consumption (one to six times per day, week, month, or year). Color photos simplify the definition of serving sizes for food items which are not consumed in normal household quantities. Furthermore, the participants are specifically asked about their use of cooking fats/oils, the frequency of consumption of sauces with meat and fish, the fat content of the milk they consume, the use of sugar and milk in coffee and tea, and the seasonal consumption of fresh fruit and vegetables. The data input and questionnaires are evaluated via the study management system (SMS) for health research, which has been developed and supervised by the Department of Epidemiology of the German Institute of Human Nutrition Potsdam-Rehbruecke (DIfE). The EPIC-FFQ is conducted at all five time points (SE, V1–V4) in both the intervention group and the control group.

#### Mediterranean Diet Adherence Screener

The Mediterranean Diet Adherence Screener (MEDAS) [28] was developed within the scope of the PREDIMED study and is an instrument used to determine the extent to which the Mediterranean dietary pattern is adhered. It comprises 14 questions. The evaluated Spanish version was translated into German for the LIBRE study and complemented by pictures of serving sizes. The German version of the MEDAS will be validated within the scope of the pilot study. The MEDAS questionnaire is conducted at all five time points (SE, V1–V4) in both the intervention and the control group and at time points V1-6 and V1-9 in the intervention group.

#### Measurement of quality of life

In order to acquire information on health-related quality of life (HRQOL), a composed questionnaire from the European Organisation for Research and Treatment of Cancer (EORTC), comprising a 30-item core questionnaire (QLQ-C30) and a 23-item breast cancer module (QLQ-BR23), is used before and after the intervention [29, 30]. Accordingly there are reference values by Scott et al. [31] which are of great importance for comparing our data. The advantage of the QLQ-C30/-BR23 is on one hand its present-day relevance and on the other hand its validation for breast cancer (QLQ-BR23). We formulate the hypothesis that regular physical activity and the implementation of healthy nutrition (intervention group) can lead to a general and specific improvement in HRQOL. The 53 questions focus on cognitive, social, emotional, and physical functioning, and one scale evaluates the global health status in general. Three other scales estimate fatigue, pain, as well as nausea and vomiting, and six single items measure dyspnea, insomnia, appetite loss, constipation, diarrhea, and financial difficulties. The breast cancer-specific module QLQ-BR23 comprises 23 multiple and single items assessing disease symptoms (breast and arm symptoms, upset caused by hair loss and systemic therapy side effects) as well as addressing body image, sexual functioning, and future perspectives. Validation studies in European countries and the USA have been conducted within a cross-cultural context [32].

#### Measurement of optimism (Life Orientation Test-Revised)

The Life Orientation Test-Revised (LOT-R) [33] is developed to collect individual differences of generalized optimism versus pessimism in the form of a personality variable. It has been applied in various intervention studies which, among other things, look at the consequences of dispositional optimism on behavior, emotion, and physical health. We also formulate the hypothesis that regular physical activity and the implementation of healthy nutrition (intervention group) can lead to a more optimistic attitude to life.

#### Measurement of chronic stress (Trier Inventory for Chronic Stress) [34]

The Screening Scale for Chronic Stress (SSCS) included in the Trier Inventory for Chronic Stress is a 12-item (short version) questionnaire which measures five different aspects of chronic stress: lack of social recognition, business pressures, social stress, chronic anxiety, and overburdening [34]. One can hypothesize that regular physical activity and the implementation of healthy nutrition (intervention group) can lead to a reduction in the individual perception of stress and to better stress management. The SSCS questionnaire has been well examined on a test statistical basis and shows good variables for quality criteria.

#### Measurement of attitudes and views on physical exercise and healthy eating

It is established in social-psychological attitude research that a person’s positive attitude towards lifestyle (here regular exercise and healthy eating) does not in itself correspond to an equivalent everyday behavior (i.e., to really eat healthfully and practice regular sports or exercise). Most people take a positive view of prevention such as cancer screening, but de facto only a small number of people actively make use of cancer prevention measures. Therefore, for the present LIBRE study, above all due to the question of the practicability of the intervention program in the sense of good compliance, it is important to ask about the attitudes towards lifestyle changes. In particular, we are interested in two questions. (1) What are the predictors of regular participation in preventive measures (sport, nutrition)? (2) Are attitudes, subjective norms, or perceived behavior control towards a behavior relevant for the intention of behavior (i.e., to train regularly and to eat healthfully) and for the de facto behavior shown? To answer these questions, we developed a new questionnaire based on the theory of planned behavior according to Fishbein and Ajzen [35]. This theory maintains that the best predictors for planned and deliberate behavior are the intentions related to behavior. Behavioral intentions are defined by attitudes towards a behavior, subjective norms, and perceived behavior control. The theory of planned behavior has been examined in many studies dealing with health psychology (e.g., surgery intentions, food adherence, and physical activity behavior among others) [36–40].

The questionnaire Evaluation of Physical Activity and Nutrition (Bewertung koerperlicher Aktivitaet und Ernaehrung, BKAE) contains questions which are divided into two sections: physical activity and regular healthy nutrition. Each section has four scales (two attitudes, subjective norms, and perceived behavior control) and two innovative single items (past behavior and factor time and guidance). All questions are measured on seven-point Likert scales and, to avoid conformational bias, several statements are reverse scaled.

### Study endpoints

The trial employs three independent co-primary endpoints: (1) the adherence to the Mediterranean diet measured by the MEDAS score, (2) the BMI, and (3) the ventilatory threshold VT1 measured in spiroergometry as a parameter of physical fitness one year after the structured intervention program.

Secondary endpoints of the study are, among others, the measurements of quality of life (EORTC QLQ-C30-/BR23), stress coping (TICS), optimism grade (LOT-R), fat calorie intake (EPIC-FFQ), maximal oxygen intake (VO_2_ max), and physical activity (IPAQ) over time (see Table 3 for details).

**Table 3:** Primary, secondary, and other outcome measures

Primary outcome measures: • Mediterranean Diet Adherence Screener (MEDAS) score • Body mass index (BMI) • Ventilatory threshold 1 (VT1) determined by bicycle spiroergometry For all three outcome measures, the change between baseline and 12 months will be analyzed. Secondary outcome measures: Psychology: • Stress coping capacity, as measured by the Trier Inventory for Chronic Stress (TICS) questionnaire • Grade of optimism, as measured by the Life Orientation Test (LOT) questionnaire • Quality of life, as measured by the European Organisation for Research and Treatment of Cancer Quality of Life Questionnaire-Core 30 (EORTC QLQ-C30) Physical activity: • Maximum oxygen consumption (VO_2_ peak), as measured by spiroergometry • Oxygen consumption (VO_2_) and watts at ventilation threshold 1 (VT1 and VT2), as measured by spiroergometry • Physical activity, as measured by the International Physical Activity Questionnaire (IPAQ) Nutrition: • Mediterranean Diet Adherence Screener (MEDAS) score • Dietary habits and calorie intake, as measured by the European Prospective Investigation into Cancer and Nutrition Study Food Frequency Questionnaire (EPIC-FFQ) • BMI Breast cancer incidence and mortality • Breast cancer incidence • Overall and breast cancer mortality rate Other outcome measures: • Attitudes and beliefs regarding physical training and healthy diet, as measured by the “Bewertung koerperlicher Aktivitaet und Ernaehrung” (BKAE) questionnaire • Satisfaction with the study • Body fat content, as measured by skinfold measurement • Tobacco and alcohol consumption • Omega 3, 6, and 9 fatty acids in the erythrocyte membrane • Serum cholesterol including serum high- and low-density lipoprotein cholesterol • Serum triglycerides, glucose, high-sensitivity C-reactive protein, and insulin • Serum proenkephalin and proneurotensin • Hospitalization days for breast cancer If appropriate, secondary and other outcome measures will be analyzed as the change from baseline over time of all available time points.	

### Sample size, randomization, and statistical analysis

The following differences of the three co-primary endpoints between study arms one year after the structured intervention are considered clinically relevant and achievable: 1 point increase in the MEDAS score, 1 kg/m^u^ reduction in BMI, 1 ml/min/kg increase in the ventilatory threshold VT1. In order to detect these differences in a two-sided *t* test with expected standard deviations as observed in the LIBRE feasibility study, a minimum of 490 evaluable patients are required with a Bonferroni adjusted significance level of 0.05/3 per single endpoint and a power of 90 %. The final sample size was set to a total of 600 patients to be enrolled to account for a drop-out rate of at least 15 %.

Randomization is performed with a 1:1 ratio using an Internet-based central randomization system based on a modified version of Pocock’s minimization algorithm, stratifying for center, disease status (no prior breast cancer, prior breast cancer without contralateral prophylactic mastectomy, prior breast cancer with contralateral mastectomy), age (<50 versus ≥50 years), physical activity as measured by the IPAQ (<24 versus ≥24 MET-h/week), BMI (<25 versus ≥25 kg/m^2^) [41].

The primary endpoints will be assessed using analysis of variance adjusting for possible differences in baseline characteristics. No interim analyses will be conducted. A written statistical analysis plan describing all planned primary and secondary endpoint analyses in detail will be set up prior to the start of data analysis.

## Discussion

The LIBRE study is one of the first prospective randomized lifestyle intervention trials involving *BRCA* mutation carriers worldwide. The purpose of this study is to demonstrate that a structured one-year exercise program combined with a Mediterranean dietary pattern will improve BMI, maximal as well as aerobic and anaerobic physical capacity, and adherence to a Mediterranean diet in *BRCA* mutation carriers. Additional secondary endpoints are to demonstrate that the lifestyle intervention will also improve psychological parameters, like quality of life, stress coping capacity, and grade of optimism. An important further intention of the trial is to investigate whether the improvement of physical fitness, body weight, quality of life, and stress coping capacity will lead to a reduction of breast cancer risk, progression of disease, and mortality. A similar approach is followed by Pasanisi et al. [42], who published a study protocol for a randomized trial in *BRCA* mutation carriers to test whether moderate calorie intake and protein restriction together with increasing physical activity will decrease serum levels of insulin-like growth factor 1 (IGF-1). They hypothesized a functional interaction between the *BRCA* genes and IGF-1 systems, which may be causal for a higher penetrance of breast cancer in those with elevated IGF-1 levels. They further hypothesize that a lifestyle intervention may reduce IGF-1 levels and thereby potentially cancer penetrance.

Both studies will evaluate the role of a lifestyle intervention program in the same cohort of *BRCA* mutation carriers. Therefore, it seems realistic to add a structured lifestyle intervention program to health care prevention programs in *BRCA* mutation carriers in the future. Current risk-reducing strategies for women at risk include prophylactic mastectomy and early detection programs. With structured exercise and dietary changes, mutation carriers will be able to independently play a role in cancer prevention, which will help to strengthen their self-government. Moreover, it is known that psychological intervention may improve stress symptoms in patients with breast cancer [15]. By improving and optimizing the women’s lifestyle, we expect a preventive health effect not only on a somatic, but also on a mental level.

A limitation of the trial might occur during randomization, especially for highly motivated participants. They may be disappointed if randomized to the control arm, and therefore may withdraw from the study or exercise nonetheless. In addition, cross-over communication regarding the Mediterranean dietary pattern between the intervention and control group cannot be completely avoided. On the other hand, it might be difficult for less motivated working women or mothers to integrate the exercise program into their everyday life.

### Trial status

The study is currently recruiting participants.

## Abbreviations

BIA, bioelectric impedance analysis; *BRCA*, breast cancer gene; CHC, coronary heart condition; ECG, electrocardiogram; GC-HBOC, German Consortium for Hereditary Breast and Ovarian Cancer; HBT, home-based training; LIBRE, Life-style intervention in *BRCA* mutation carriers; LOT-R, Life of Orientation Test; MD, Mediterranean dietary pattern; MET, Metabolic Equivalent of Task; QLQ-C30/-BR23, Quality of Life Questionnaire-Core 30 and breast cancer module 23; SE, study entry; SSCS, Screening Scale for Chronic Stress; V, visit

## Additional files

Additional file 1: SPIRIT checklist. (PDF 262 kb) Additional file 2: SPIRIT flow diagram of the LIBRE study. (PDF 91 kb)
