# Supplementary material for: Effects of lifestyle intervention in BRCA1/2 mutation carriers on nutrition, BMI, and physical fitness (LIBRE study): study protocol for a randomized controlled trial
Source: Trials. 2016 Jul 29;17:368. doi: 10.1186/s13063-016-1504-0 (PMC4966818; doi:10.1186/s13063-016-1504-0)
Supplement: Additional file 1: — SPIRIT checklist. (PDF 262 kb) [file 13063_2016_1504_MOESM1_ESM.pdf]

SPIRIT Checklist

ADMINISTRATIVE INFORMATION

1 Title

Effects of lifestyle intervention in BRCA1/2 mutation carriers on nutrition, BMI and physical fitness (LIBRE Study): study protocol for a randomized, controlled trial.

2 Trial Registration

2a Clinical Gov

ClinicalTrials.gov Identifier: NCT02516540:
http://www.clinicaltrials.gov/ct2/show/NCT02516540?term=LIBRE&rank=1

2b WHO

World Health Organization Trial Registration:
http://apps.who.int/trialsearch/Trial2.aspx?TrialID=NCT02516540
The WHO trial registration data set is attached to the appendices.

3 Protocol Version

Version 01, dated 2015/06/26

4 Funding

The trial is funded by German Cancer Aid (Deutsche Krebshilfe: http://www.krebshilfe.de) within the Priority Program "Primary Prevention of Cancer" (Grant no. 110013). The awarded grant comes from a major charitable funding body in Germany.
Deutsche Krebshilfe (German Cancer Aid),
http://www.krebshilfe.de/metanavigation/english.html
Stiftung Deutsche Krebshilfe, Buschstr. 32, D-53113 Bonn, Germany
Phone: +49 228 729900, Fax: +49 228 7299011

5 Roles and Responsibilities

5a Protocol contributors

Principal Investigator	Prof. Dr. med. Marion Kiechle, MD<br>Klinikum rechts der Isar<br>Technische Universität München<br>Ismaningerstrasse 22, D- 81675 München<br>T +49 89 41402420<br>F +49 89 41404831<br>marion.kiechle@lrz.tum.de
Project Management	Daniela Schemmer<br>Klinikum rechts der Isar<br>Technische Universität München

	Ismaningerstrasse 22, D- 81675 München<br>T.: +49 89 41402433<br>F: +49 89 41404846<br>Daniela.schemmer@mri.tum.de
Sport Medicine	Prof. Dr. med. Martin Halle, MD<br>Technische Universität München<br>Arcisstr. 21, D-80333 München<br>T +49 89 28924431<br>F +49 89 28924450<br>halle@tum.de
Nutritional Medicine	Prof. Dr. med. Stephan C. Bischoff, MD<br>Universität Hohenheim, Stuttgart<br>Fruwirthstr. 12, D-70593 Stuttgart<br>T +49 711 45924101<br>F +49 711 45924343<br>bischoff.stephan@uni-hohenheim.de
Psychology	Prof. Dr. med. Michael Siniachtkin, MD<br>Universitätsklinikum Schleswig-Holstein, Campus Kiel,<br>Diesterwegstrasse 10-12, D-24113 Kiel<br>T: +49 431 6594630<br>F: +49 431 6594639<br>siniatchkin@med-psych.uni-kiel.de
Statistics / Data Management	Prof. Dr. med. Markus Löffler, MD<br>PD Dr. med. habil. Christoph Engel, MD<br>University of Leipzig<br>Institute for Medical Informatics, Statistics and Epidemiology<br>Haertelstraße 16-18, 04107 Leipzig, Germany<br>T +49 341 9716100<br>F +49 341 9716109<br>markus.loeffler@imise.uni-leipzig.de<br>christoph.engel@imise.uni-leipzig.de

5b Trial sponsor

Technical University Munich, Klinikum Rechts der Isar, Ismaninger Strasse 22, 81675 Munich, Germany, http://www.mri.tum.de

5c Role of study sponsor and funders

The trial sponsor accepts full responsibility for the entire trial. The funder has no authority and is not involved in the following activities: study design; collection, management, analysis, and interpretation of data; writing of the report; and the decision to submit the report for publication.

5d Steering committee

The steering committee consists of the protocol contributors (see 5a). It is responsible for trial coordination, endpoint adjudication and data analysis together with the team responsible for statistics and data management.

INTRODUCTION

6 Background and Rationale

6a Women with highly penetrant BRCA mutations have a 55-60% risk for breast cancer and a 16-59% risk of developing ovarian cancer. However, since penetrance rates are not 100%, it can be postulated that risk-modulating factors do exist. It could be shown that the risk of development of cancer in gene carriers may be influenced through genetic factors (polymorphisms) as well as exogenous factors such as number of pregnancies, year of birth, and physical activity during youth. The risk for breast cancer is lower if gene carriers were born before 1940, have given birth or were physically active during their youth.

The risk for developing sporadic breast cancer is considerably influenced by physical activity, nutrition and body weight and these factors also affect disease progression. Likewise, it has been demonstrated in several prospective studies that regular physical activity can significantly reduce breast cancer incidence in post and premenopausal women, the risk being reduced on average by 25%. Furthermore the risks of recurrence and mortality in women with breast cancer are reduced by 50% if they engage in regular physical activity. Further advantages of physical activity include a gain in quality of life, increased fitness and better tolerance of chemotherapy. Nutrition also influences the risk for breast cancer. Obesity and weight gain increase the risk of breast cancer in both pre- and postmenopausal subjects. A weight gain of more than 20 kg after the age of 18 doubles the risk for breast cancer. Furthermore women with a BMI of >30 kg/m^2 have a greater risk of developing distant metastases and of dying of breast cancer. In a prospective study with sporadic breast cancer patients who were given adjuvant standard therapy, a calorie and fat reduced nutrition program led to a significant improvement in recurrence rate.

Further risk factors for breast cancer include depression, a pessimistic outlook on life, and problems coping with stress. It was shown that physical activity has a favorable influence on stress management and on depression. Many studies have convincingly documented the great significance of an optimistic life perspective for different psychological and somatic disorders. A positive correlation was shown between an optimistic outlook on life and psychological well-being, health, stress reduction and mortality, as well as a quicker recovery rate.

So far no studies exist in this context on women with hereditary breast cancer or women with a deleterious BRCA mutation. Even retrospective data is rare. There is only one publication on this subject by Manders in 2011, which reports on an association between increased body weight and an increased risk for breast cancer in BRCA1/2 mutation carriers.

We therefore aim to examine, whether a lifestyle intervention in the form of structured physical endurance training and nutrition education, emphasizing the Mediterranean dietary

pattern, will lead to an improvement of nutritional behavior (adherence to the Mediterranean diet), BMI, physical fitness, quality of life, and optimistic outlook on life, and cause a significant reduction of the perceived stress. Secondary aims of the study are to investigate whether the intervention will lead to a reduction of breast cancer incidence and breast cancer mortality in BRCA1 and BRCA2 mutation carriers.

The most important studies in this field are listed as follows:

Antoni MH, Lutgendorf SK, Blomberg B, Carver CS, Lechner S, Diaz A et al. Cognitive-behavioral stress management reverses anxiety-related leukocyte transcriptional dynamics. *Biol.Psychiatry* 2012;71:366-72.

Antoni MH, Lechner S, Diaz A, Vargas S, Holley H, Phillips K et al. Cognitive behavioral stress management effects on psychosocial and physiological adaptation in women undergoing treatment for breast cancer. *Brain Behav.Immun.* 2009;23:580-91.

Backman M, Wengstrom Y, Johansson B, Skoldengen I, Borjesson S, Tarnbro S et al. A randomized pilot study with daily walking during adjuvant chemotherapy for patients with breast and colorectal cancer. *Acta Oncol.* 2014;53:510-20.

Bissonauth V, Shatenstein B, Fafard E, Maugard C, Robidoux A, Narod S et al. Weight History, Smoking, Physical Activity and Breast Cancer Risk among French-Canadian Women Non-Carriers of More Frequent BRCA1/2 Mutations. *J Cancer Epidemiol.* 2009;2009:748367.

Chlebowski RT, Blackburn GL, Thomson CA, Nixon DW, Shapiro A, Hoy MK et al. Dietary fat reduction and breast cancer outcome: interim efficacy results from the Women's Intervention Nutrition Study. *J Natl.Cancer Inst.* 2006;98:1767-76.

Culver JL, Arena PL, Antoni MH, Carver CS. Coping and distress among women under treatment for early stage breast cancer: comparing African Americans, Hispanics and non-Hispanic Whites. *Psychooncology.* 2002;11:495-504.

Courneya KS, McKenzie DC, Gelmon K, Mackey JR, Reid RD, Yasui Y et al. A multicenter randomized trial of the effects of exercise dose and type on psychosocial distress in breast cancer patients undergoing chemotherapy. *Cancer Epidemiol.Biomarkers Prev.* 2014;23:857-64.

Eliassen AH, Hankinson SE, Rosner B, Holmes MD, Willett WC. Physical activity and risk of breast cancer among postmenopausal women. *Arch.Intern.Med.* 2010;170:1758-64.

Ewertz M, Jensen MB, Gunnarsdottir KA, Hojris I, Jakobsen EH, Nielsen D et al. Effect of obesity on prognosis after early-stage breast cancer. *J Clin.Oncol.* 2011;29:25-31.

Friedenreich CM, Woolcott CG, McTiernan A, Ballard-Barbash R, Brant RF, Stanczyk FZ et al. Alberta physical activity and breast cancer prevention trial: sex hormone changes in a year-long exercise intervention among postmenopausal women. *J Clin.Oncol.* 2010;28:1458-66.

Holmes MD, Chen WY, Feskanich D, Kroenke CH, Colditz GA. Physical activity and survival after breast cancer diagnosis. *JAMA* 2005;293:2479-86.

Manders P, Pijpe A, Hooning MJ, Kluijt I, Vasen HF, Hoogerbrugge N et al. Body weight and risk of breast cancer in BRCA1/2 mutation carriers. *Breast Cancer Res.Treat.* 2011;126:193-202.

Pettapiece-Phillips R, Narod SA, Kotsopoulos J. The role of body size and physical activity on the risk of breast cancer in BRCA mutation carriers. *Cancer Causes Control* 2015;26:333-44.

Phillips KM, Antoni MH, Lechner SC, Blomberg BB, Llabre MM, Avisar E et al. Stress management intervention reduces serum cortisol and increases relaxation during treatment for nonmetastatic breast cancer. *Psychosom.Med.* 2008;70:1044-9.

Rosato V, Bosetti C, Talamini R, Levi F, Montella M, Giacosa A et al. Metabolic syndrome and the risk of breast cancer in postmenopausal women. *Ann.Oncol.* 2011;22:2687-92.

Wimberly SR, Carver CS, Antoni MH. Effects of optimism, interpersonal relationships, and distress on psychosexual well-being among women with early stage breast cancer. *Psychol.Health* 2008;23:57-72.

6b Choice of comparators

The control group receives standard publicly available information on a healthy nutrition according to national guidelines (German society of nutrition) and the positive effect of exercise on the health status.

7 Objectives

The trial has three independent co-primary endpoints: 1) the adherence to the Mediterranean diet as measured by the MEDAS score 2) the BMI and 3) the ventilatory threshold VT1 measured in spiroergometry as a parameter of physical fitness one year after the structured intervention program.
Secondary endpoints of the study are amongst others the measurements of quality of life, stress coping, optimism grade, fat calorie intake, maximal oxygen intake (VO2max), and physical activity over time.

8 Trial design

Multicenter, prospective, two-armed randomized (1:1) controlled clinical trial.

METHODS: PARTICIPANTS, INTERVENTIONS, AND OUTCOMES

9 Study setting

The study is conducted in 18 German hospitals, including 15 study centers which are members of the German Consortium of Hereditary Breast and Ovarian Cancer (GC-HBOC). The study sites are listed on the homepage of the GC-HBOC (www.konsortium-familiaerer-brustkrebs.de).

10 Eligibility criteria

Inclusion criteria for participants (all criteria must apply):

- Women with a pathogenic *BRCA1* or *BRCA2* germline mutation
- Age over 18 years
- Written informed consent

Exclusion criteria:

- Ongoing chemo- or radiation therapy (recruitment is possible six weeks after completing therapy)
- Metastatic tumor disease
- Expectancy of life < 3 years
- Limited cardiovascular and lung diseases (instable CHC, heart failure stage IV, COPD GOLD IV, maximal blood pressure at rest > 160/100 mmHg)
- Significant orthopedic problems, not allowing exercise
- Serious diseases, not allowing a participation in group interventions (e.g. psychiatric or internal ailment)
- Karnofsky-Index < 60%
- Women with an exercise capacity < 50 Watt
- Food allergies not allowing consumption of a Mediterranean dietary pattern
- Vegans
- BMI < 15 kg/m²
- Pregnancy
- Insufficient knowledge of the German language
- Lack of Compliance
- Current participation in other lifestyle intervention trials

Eligibility criteria for study centers and individuals who will perform the interventions:

Study centers have to ensure the relevant expertise and personnel resources in order to conduct the medical care, nutritional intervention and supervised training according to standard operation procedures (SOPs). Study centers have to provide the technical equipment to guarantee a laboratory testing at the center and a shipment of blood specimen to the central blood analysis center in Hohenheim within 24 hours, as required in the SOP. For electronic data capture, personal computers with internet access are needed.

11 Interventions

11a Interventions for each group

Intervention Group:

A lifestyle intervention program lasting 12 months is adopted in the intervention group. The program is applied intensively during the first 3 months and is maintained and monitored for the following 9 months through monthly contacts and meetings. The lifestyle intervention program comprises the following measures:

Physical activity: The LIBRE training program is primarily endurance-oriented training, which is completed during the course of 1 year. After a mandatory introductory lecture on the theory behind the intervention training, the goal is to increase physical activity to ≥18 MET-h/week (MET= Metabolic Equivalent Task). This activity level has been correlated

consistently with a reduction of morbidity and mortality in breast cancer patients [1,2]. This goal should be achieved within the first 12 weeks and maintained throughout the whole study period. Each subject receives an individual training plan, which is continuously adapted according to her fitness status to support this goal. The training is divided into two phases: the intensity of the first phase should be at least 50-60% of peak oxygen consumption (VO2peak) (initial phase, weeks 1-6) and 60-75% of the VO2peak (optimization phase, weeks 7-12). In the first 12 weeks of the intervention program, training takes place 2x/week as supervised training and 1x/week as home-based training (HBT). Afterwards supervised training units are only carried out monthly so that training in phase II (months 4-12) is mainly HBT. Training continues to take place with an intensity of at least 60-75% of the VO2peak in accordance with the individual training plan.
A record of compliance with the training intervention program as well as achieved MET*h/week is kept in the training diaries (questionnaire on physical activity, V0-V2). Participants are asked to record their daily activities as well as intentional physical activity including intensity and duration of training. The training intensity levels are assessed from spiroergometry and are outlined in the diary, which facilitates training control. Monthly-supervised training units (as of V1) offer the opportunity to realign training intensity and discuss any problems with adherence to training. In addition, physical activity is recorded by questionnaires (IPAQ 21,22 from SE-V4 and physical fitness is assessed by cardiopulmonary exercise testing by spiroergometry (VO2peak) 23 at time points SE, V1 und V2.
Nutrition: Within the framework of the LIBRE study, the nutrition intervention is based on the principles of a Mediterranean dietary pattern (MD). Furthermore, obese patients (BMI>30 kg/m2) are instructed to limit their energy (kcal) intake. Nutrition intervention in the intervention group begins with an intensive three months' nutrition program during which bi-weekly nutrition courses led by dietitians take place in groups. The group course includes a cooking class and guided tour of a supermarket. At the end of the first three months, the nutrition courses take place at monthly intervals for the remaining course of the first study year. The main objective of the nutrition intervention is to provide practical nutritional training, which should enable the subjects to achieve a long term change in their eating habits, replacing former eating habits with the MD. Eating habits are recorded using validated questionnaires (EPIC-FFQ and MEDAS questionnaires) at the time points SE, V1, V2, V3 and V4. Participants of the intervention group additionally received the MEDAS questionnaire at time points V1-6 and V1-9.
Psychological support: Psychological support of the intervention group comprises solely of an explanation of the psychological data survey questionnaires given to the study participants. Explicit psychological support is not planned. The participants are informed that the objective of the lifestyle study is, among other things, that the lifestyle change including regular physical activity and healthy eating should lead to an improvement in general quality of life, stress reduction, a more optimistic outlook on life and better attitude towards endurance training and MD. In order to verify this, the participants receive questionnaires (TICS, LOT-R, EORTC QLQ-C30/-BR23 and BKAE) at different time points (SE, V1, V2, V3 and V4). Advice on psycho-oncological aspects of the LIBRE study is given during the introduction

lecture at the beginning of the study. Particular importance is attached to participants giving as detailed information as possible in the survey questionnaires.
Psychological advice given to the study participants should serve to inform the subjects of the significance and improvement of psycho-social lifestyles for the prevention of breast and ovarian cancer, as well as to promote compliance and recognize possible psychological impediments for a participation in the study.

1.Courneya KS, McKenzie DC, Mackey JR, et al. Subgroup effects in a randomised trial of different types and doses of exercise during breast cancer chemotherapy. British journal of cancer 2014;111:1718-25.
2.Holmes MD, Chen WY, Feskanich D, Kroenke CH, Colditz GA. Physical activity and survival after breast cancer diagnosis. Jama 2005;293:2479-86.

Control Group
The control group will receive a mandatory introduction lecture on the positive effects of physical activity on the incidence and prognosis of breast cancer. Afterwards all participants will be given a brochure providing the most important facts on this topic.
Contrary to the intervention group, no training and no physical activity diaries will be provided. Changes in physical activity behavior are measured identically to the interventional group through questionnaires (IPAQ SE-V4) and examinations of physical capacity (VO2peak) at the time points SE, V1 and V2.
Additionally, a dietitian led nutrition group lesson on healthy eating will be held for the control group. During this lesson the subjects receive general information based on the recommendations of the German Society of Nutrition, which is referred to as "usual care in Germany" in this study. Eating habits will be recorded identically to the intervention group via validated questionnaires (EPIC-FFQ und MEDAS-questionnaire) at the defined time points SE, V1, V2, V3, V4.
The psychological guidance of the control group consists of the explanation of the psychological data entry forms, which are given to the study participants. An explicit psycho-oncological intervention strategy is also not intended in this group. The participants will be informed that changes in daily routine concerning physical activity and dietary habits amongst other things should resolve in improvement of quality of life, a reduction of stress, and a more optimistic approach to future life. In order to verify this, the participants will receive questionnaires (TICS, LOT-R, EORTC QLQ-C30/-BR23 and BKAE for registration of changes in attitude towards physical activity and MD) at SE, V1-V4. The psychological information of the control group corresponds to those of the intervention group.

11b Criteria for discontinuation of an individual patient

Withdrawal of consent, excessive intolerance to the intervention, or decision made by the trial physician.

11c Strategies to improve adherence to intervention protocols, and any

procedures for monitoring adherence

Monthly-supervised training units will ensure to realign training intensity and discuss any problems with adherence to training. In addition, motion sensors (accelerometry) and physical activity diaries are expected to improve adherence to physical training. Adherence to the nutritional intervention is reinforced by the regularity of nutrition courses, including repetition of contents, and the possibility for telephone calls. Attendance lists supervise adherence of participants to nutrition classes. Adherence to the intended activity behavior and to dietary recommendations can be monitored via questionnaires, clinical examination, spiroergometry and laboratory tests at the defined time points V1, V2, V3, V4.

11d Relevant concomitant care and interventions that are permitted or prohibited during the trial

Participation in any other intervention trial is not allowed.

12 Outcomes

Primary Outcome Measures:

- Mediterranean Diet Adherence Screener (MEDAS) Score
- Body mass index (BMI)
- Ventilatory threshold 1 (VT1) as determined by bicycle spiroergometry

For all three outcome measures, the change between baseline and 12 months will be analyzed.

Secondary Outcome Measures:

Psychology:

- Stress coping capacity, as measured by the Trier Inventory for Chronic Stress (TICS) questionnaire
- Grade of optimism, as measured by the Life Orientation Test (LOT) questionnaire
- Quality of life, as measured by the European Organisation for Research and Treatment of Cancer Quality of Life Questionnaire-Core 30 (EORTC QLQ-C30)

Physical activity:

- Maximum oxygen consumption (VO_2peak), as measured by spiroergometry
- Oxygen consumption (VO_2) and Watts at ventilation threshold 1 (VT1 and VT2), as measured by spiroergometry
- Physical activity, as measured by the International Physical Activity Questionnaire (IPAQ)

Nutrition:

- Mediterranean Diet Adherence Screener (MEDAS) Score
- Dietary habits and calory intake, as measured by the European Prospective Investigation into Cancer and Nutrition Study Food Frequency Questionnaire (EPIC-FFQ)
- Body mass index (BMI)

Breast cancer incidence and mortality

- Breast cancer incidence
- Overall and breast cancer mortality rate

Other Outcome Measures:
- Attitudes and beliefs regarding physical training and healthy diet, as measured by the "Bewertung koerperlicher Aktivitaet und Ernaehrung" (BKAE) questionnaire
- Satisfaction with the study
- Body fat content, as measured by skin folds measurement
- Tobacco and alcohol consumption
- Omega 3, 6 and 9 fatty acid in the erythrocyte membrane
- Serum cholesterol including serum high and low density lipoprotein cholesterol
- Serum triglycerides, glucose, high sensitivity c-reactive protein and insulin
- Serum proenkephalin and proneurotensin
- Hospitalisation days for breast cancer

If appropriate, secondary and other outcome measures will be analyzed as the change from baseline over time of all available time points.

13 Participant timeline

The trial consists of a 12-week intensive intervention treatment phase followed by 9 months of light intervention and two years of follow-up. The total trial period is 36 months. The participants are screened for the inclusion criteria, sign the declaration of consent and are randomized to either the control or intervention group at time point SE (study entry). At this study date the participants are examined at the study centers and fill out questionnaires concerning their medical history, social background, psychological health and current physical activity and nutritional habits.
V0 is the start of the intervention and is scheduled not longer than 3 months after SE. The control group receives general information about a healthy lifestyle and cancer risk at the beginning of the study. The intervention group takes part in a 12-months intervention program including a structured physical exercise program three times a week and a nutrition training. Participants of the intervention group are asked to fill out the MEDAS questionnaire at the time points V1-6 and V1-9.
All patients undergo a regular clinical examination including blood testing, EKG, clinical questionnaires and anthropometry at time points V1, V2, V3 and V4. Spiroergometry is conducted at time point SE, V1 and V2.
The timeline is summarized in Table 1.

Table 1: Schedule of enrolment, interventions, and assessments.

	Enrolment	Start						Close-out	Follow up
**TIMEPOINT****	***SE***	**V0**	***V1 (3Mo)***	***6Mo***	***9Mo***	***V2 (12Mo)***	***V3 (24Mo)***	***V4 (36Mo)***	***+12Mo***
**ENROLMENT:**									
**Eligibility screen**	X								
**Informed consent**	X								
**Randomisation**	X								
**INTERVENTIONS:**									
[Interventiongroup "I"]	X	Intervention phase V0 – V2 ⟷					X	X	X
[control group "C"]	X	X	X	X	X	X	X	X	X
**ASSESSMENTS:**									
Clinical Baseline	C+I								
Clinical follow up						C+I	C+I	C+I	C+I
Spiroergometry	C+I		C+I			C+I	C+I	C+I	
Accelerometry	C+I		C+I	C+I	C+I	C+I			
Questionnaire "training" (IPAQ-L)		C+I	C+I	C+I	C+I	C+I	C+I	C+I	
Questionnaires "nutrition"	C+I		C+I			C+I	C+I	C+I	
Lab	C+I		C+I			C+I	C+I	C+I	
Clinical examination	C+I		C+I			C+I	C+I	C+I	
Anthropometry	C+I		C+I			C+I	C+I	C+I	
"Psychological" questionnaires (EORTC QLQ-C30/ BR23,TICS,BKAE, LOT-R)	C+I		C+I			C+I	C+I	C+I	

C= control group; I= Intervention group, SE= study entry

14 Sample size

The following differences of the three co-primary endpoints between study arms one year after the structured intervention are considered clinically relevant and achievable: 1 point increase in the MEDAS score, 1 kg/m2 reduction in BMI, 1 ml/min/kg increase in the ventilatory threshold VT1. In order to detect these differences in a two-sided t-test with expected standard deviations as observed in the LIBRE feasibility study, a minimum of 490 evaluable patients are required with a Bonferroni adjusted significance level of 0.05/3 per single endpoint and a power of 90%. The final sample size was set to a total of 600 patients to be enrolled to account for a drop-out rate of at least 15%.

15 Recruitment

Most of the trial sites are members of the German Consortium of Hereditary Breast and Ovarian Cancer with a long-standing experience in counseling members of families suspected of having hereditary breast and ovarian cancer. All patients to be enrolled in this trial are already under clinical care in the trial sites.

METHODS: ASSIGNMENT OF INTERVENTIONS

Allocation

16a Sequence generation

Randomization is performed with an 1:1 ratio using Pocock's minimization algorithm, stratifying for center, disease status (no prior breast cancer, prior breast cancer without contralateral prophylactic mastectomy, prior breast cancer with contralateral mastectomy), age (<50 vs. ≥50 years), physical activity as measured by the IPAQ questionnaire (<24 vs. ≥24 MET h/week), BMI (<25 vs. ≥25 kg/m2).

16b Allocation concealment mechanism

The randomization is performed centrally using an internet-based central randomization system.

16c Implementation

The allocation sequence is generated centrally by the team responsible for statistics and data management. The trial participants are enrolled by physicians at the recruiting trial sites.

17a Blinding

The trial is not blinded.

17b Unblinding

Not applicable.

METHODS: DATA COLLECTION, MANAGEMENT AND ANALYSIS

18 Data collection methods

18a Plans for assessment and collection of outcome, baseline, and other trial data

Primary outcomes: The criteria for the three independent co-primary endpoints: 1) the adherence to the Mediterranean diet measured by the MEDAS score 2) the BMI and 3) the ventilatory threshold VT1 measured in spiroergometry as a parameter of physical fitness one year after the structured intervention program are derived from other large intervention studies. The randomization and the monitoring are carried out at each study center. Deviations are documented in the central database.

Secondary outcomes: The secondary endpoints quality of life, stress coping, optimism grade, physical activity and fat calorie intake are measured by the validated questionnaires EORTC QLQ-C30-/BR23, TICS, LOT-R, IPAQ and EPICFFQ. Changes in Body Mass Index (BMI) and maximal oxygen intake (VO_2max) are measured during the physical examination and the spiroergometry and are compared to a healthy standard population. The assessment will be performed after 3, 12, 24 and 36 months of intervention at each study center and will be compared to the baseline measurements at study entry.
Each center's personnel is trained in the study requirements, the key messages of the lifestyle intervention and the use of the central database. The dietitian and the trainer get specifically instructed. The results of the spiroergometric testing are collected and checked at the study coordination center in Munich.

Standard operating procedures (SOPs) have been defined for medical examinations, the nutritional intervention, nutritional counseling, blood sample taking and preparation, spiroergometry, anthropometry, pedometry and the center-based training units.

18b Plans to promote participant retention and complete follow-up

All participants are informed about the importance of complete datasets and follow-up. The participants of the intervention group are constantly reminded of the key messages of the lifestyle intervention during study dates and via e-mail / telephone, if they miss a study date. All patients get an expense allowance after completion of V1.
The participants receive feedback after their clinical examination, laboratory testing and fitness test on how to maintain their fitness level or how to improve this and are encouraged to continue the training.

To measure the patients' satisfaction and to figure out difficulties, participants are invited to fill out a satisfaction questionnaires at the time points V1, V2, V3 and V4.

Prior to the final data collection, the participants receive a reminder to send back missing

questionnaires to guarantee complete data sets.

If participants deviate from intervention protocols, the reasons have to be carefully documented and the participant should be motivated to catch up on the intervention protocol. The dataset should be collected as before.

19 Data Management

All trial data will be collected at the study centers and entered using the browser-based online data capture and management system 'OpenClinica' (https://www.openclinica.com/). All trial data is centrally stored in a database at the center responsible for statistics and data management of the trial. Data is checked centrally for completeness, plausibility and consistency (e.g. range checks). Problems with data quality are reported back to the trial sites.

20 Statistical methods

20a Statistical methods

The primary endpoints will be assessed using analysis of variance adjusting for possible differences in baseline characteristics. No interim analyses will be conducted. A written statistical analysis plan describing all planned primary and secondary endpoint analysis in detail will be set up prior to the start of data analysis.

20b Methods for any additional analyses

Subgroup analyses for secondary endpoints are planned for patients who have or have not successfully completed the intervention, and for patients with our without breast cancer before study inclusion.

20c Definition of analysis population relating to protocol non-adherence

The primary endpoint analysis comprises all randomized patients. Per-protocol analyses will be conducted for patients defined as having successfully completed the intervention. No imputations will be made for missing data.

METHODS: MONITORING

21 Data monitoring

21a Composition of data monitoring committee (DMC)

A DMC will not be implemented.

21b Description of any interim analyses and stopping guidelines, including who will have access to these interim results and make the final decision to terminate the trial

No interim analyses will be conducted.

22 Harms

Adverse events (AE) are defined as all unfavorable medical events. This includes disease, clinical symptoms and pathological laboratory testing that come up or aggravate after the inclusion in the clinical trial and especially after the inclusion in the intervention system.
An event is classified as severe adverse event (SAE) following the ICH guideline E2A, article IIb) if it i) leads to death, ii) is life-threatening, iii) leads to hospital treatment or to an extension of a current hospital treatment, iv) leads to permanent damages, or v) is a congenital malformation or a birth defect. An adverse event is defined as unexpected, if it is not yet described as caused by this form of intervention or if the manifestation form or intensity is not yet described in literature. AEs have to be documented from the beginning up to the completion of the intervention phase on a special documentation form. The occurrence of any SAE has to be reported to the PI immediately (within 24 hours) by the investigator. The report of the investigator has to be checked for completeness and plausibility, if needed further questions have to be proceeded. Callbacks are planned in the following frequency: 2 weeks after the report of an SAE, 2 weeks after the first callback, 1 week after the second callback. In case of missing data regarding outcome and end date after the third callback, further callbacks are planned in larger time intervals. If necessary, the PI should be involved. The event is closed, if the patient's outcome is defined as „recovered" „recovered with sequel" or „fatal" and the end date is documented. As an exception, the PI can decide to close an event without the information mentioned above. If the participant needs a therapeutic intervention due to an AE, it has to be carried out, following the guidelines of modern medicine to recover the patient's health. Appropriate drugs and instruments should be held ready if resuscitation procedures should be needed. The treatment of an AE / SAE has to be documented.

23 Auditing

Auditing procedures are not implemented.

ETHICS AND DISSEMINATION

24 Research ethics approval

The study is conducted in accordance with the the Declaration of Helsinki of 1996 and the German medical professional codex as well as the German Federal Data Protection Act (BDSG). The trial has been registered at ClinicalTrials.gov (reference: NCT02516540).
All women participate in the study voluntarily and give written informed consent prior to study begin. They are informed that they can withdraw their consent and stop participation at any time without disclosing the reasons and without negative consequences for their future medical care. The responsible ethics review boards of all participating trial sites approved the study protocol (Reference number 5686/13 for the leading vote of the Klinikum Rechts der Isar of the Technical University of Munich).

25 Protocol amendments

The ethics review board will be informed about all study protocol modifications and asked for approval. Trial registry entries will be updated accordingly.

26 Consent or assent

26a Who will obtain informed consent or assent from potential trial participants or authorized surrogates, and how (see Item 32)

Physicians at the study centre introduce the trial to appropiate patients and collect their personal data. Patients receive patient information in paper form and have the possibility to discuss these with a physician at the study centre before they sign the declaration of consent and inclusion in the study.

26b Additional consent provisions for collection and use of participant data and biological specimens in ancillary studies, if applicable

No additional consent for collection and use of participant data and biological specimens in ancillary studies is necessary.

27 Confidentiality

Personal information about participants will be shared only between the involved physician, trainer and dietitian. No personal information (i.e. patient names) will be stored in the central trial database. All medical information will be stored centrally using pseudonyms. This applies also to all specimens. A matching list between the study pseudonym and the personal information is only available to the involved trial personnel at the study site. Only the trial personnel has access to the central documentation system.

28 Declaration of interests

The principal investigator and all other protocol contributors declare that they have no financial and competing interests.

29 Access to data

Data is kept at the center responsible for biostatistics and data management and will only be made available to staff members carrying out statistical analysis as approved by the steering board of the study.

30 Ancillary and post-trial care

Study participants receive an expense allowance of €80 in the control group and €100 in the intervention group to cover travel costs. The allowance is paid after time point V1.
Accidents during study dates or study-related physical exercise and accidents that occur on the way to study dates, are covered by a group insurance up to € 52,000 per participant.

HDI Versicherung AG
HDI-Platz 1
30659 Hannover
Insurance number: 22-010537956-2

31 Dissemination policy

31a Trial results will be published to healthcare professionals and the scientific community as publications in peer-reviewed scientific journals or as oral presentation or posters at scientific conferences, nationally and internationally. In addition, results will be transported to the public via public relations office of the university (TUM) and university hospital (Klinikum rechts der Isar).

31b Authorship eligibility criteria will follow the guidelines of The International Committee of Medical Journal Editors (ICMJE) evolved from the ***Vancouver Group,*** which published its first requirements for manuscripts in 1978. Currently it is not planned to involve a professional writer.

31c After completion of the study and publication of the results, data and material will be provided on request to the scientific community.

APPENDICES

32 Informed consent materials

The patient information and the patient consent form are attached (German language)

33 Biological specimens

Not applicable.

34 WHO Trial registration

The WHO trial registration is attached.
