# Supplementary material for: Effects of lifestyle intervention in BRCA1/2 mutation carriers on nutrition, BMI, and physical fitness (LIBRE study): study protocol for a randomized controlled trial
Source: Trials. 2016 Jul 29;17:368. doi: 10.1186/s13063-016-1504-0 (PMC4966818; doi:10.1186/s13063-016-1504-0)
Supplement: Additional file 2: — SPIRIT flow diagram of the LIBRE study. (PDF 91 kb) [file 13063_2016_1504_MOESM2_ESM.pdf]

SPIRIT Flow Diagram of the LIBRE Study

	Enrolment	Start						Close-out	Follow up
**TIMEPOINT****	***SE***	**V0**	***V1 (3Mo)***	***6Mo***	***9Mo***	***V2 (12Mo)***	***V3 (24Mo)***	***V4 (36Mo)***	***+12Mo***
**ENROLMENT:**									
**Eligibility screen**	X								
**Informed consent**	X								
**Randomisation**	X								
**INTERVENTIONS:**									
[Interventiongroup "I"]	X	Intervention phase V0 – V2 (←→)					X	X	X
[control group "C"]	X	X	X	X	X	X	X	X	X
**ASSESSMENTS:**									
Clinical Baseline	C+I								
Clinical follow up						C+I	C+I	C+I	C+I
Spiroergometry	C+I		C+I			C+I	C+I	C+I	
Accelerometry	C+I		C+I	C+I	C+I	C+I			
Questionnaire "training" (IPAQ-L)		C+I	C+I	C+I	C+I	C+I	C+I	C+I	
Questionnaires "nutrition"	C+I		C+I			C+I	C+I	C+I	
Lab	C+I		C+I			C+I	C+I	C+I	
Clinical examination	C+I		C+I			C+I	C+I	C+I	
Anthropometry	C+I		C+I			C+I	C+I	C+I	
"Psychological" questionnaires (EORTC QLQ-C30/ BR23,TICS,BKAE, LOT-R)	C+I		C+I			C+I	C+I	C+I	

C= control group; I= Intervention group, SE= study entry
